# Self-managed physical activity in breast cancer survivors: A scoping review

**DOI:** 10.1371/journal.pone.0284807

**Published:** 2023-04-24

**Authors:** Maria Chiara Bò, Andrea Merlo, Maria Bernadette Ligabue, Maria Chiara Bassi, Mirco Lusuardi, Isabella Campanini

**Affiliations:** 1 LAM–Motion Analysis Laboratory, Neuromotor and Rehabilitation Department, Azienda USL-IRCCS di Reggio Emilia, San Sebastiano Hospital, Correggio (Reggio Emilia), Italy; 2 Merlo Bioengineering, Parma, Italy; 3 Motor Rehabilitation Unit, Neuromotor and Rehabilitation Department, Azienda USL-IRCCS di Reggio Emilia, San Sebastiano Hospital, Correggio (Reggio Emilia), Italy; 4 Medical Library, Azienda USL-IRCCS di Reggio Emilia, Correggio (Reggio Emilia), Italy; 5 Neuromotor and Rehabilitation Department, Azienda USL-IRCCS Reggio Emilia, Correggio (Reggio Emilia), Italy; Pozan University of Physical Education, POLAND

## Abstract

**Objective:**

Breast cancer survivors (BCS) experience many issues of rehabilitative concern due to the treatments they have undergone. Given the chronicity of these outcomes, the increasing number of survivors, and the positive results obtained by supervised exercise, professionals should consider offering self-managed physical activity (PA) programs to this population. Our aim was to map the currently available evidence about self-care rehabilitation for BCS.

**Methods:**

Medline, CINAHL, and Cochrane databases were searched for primary literature. Scoping review methodological frameworks were used to tackle the heterogeneity of the topic. Studies investigating self-managed PA interventions prescribed to adult BCS were included.

**Results:**

One hundred-eight studies were included, with sample sizes ranging from 6 to 692 patients. Information was systematically collected in tables displaying study design, type of PA, duration and recommended frequency, professional leading the study, type of supervision, initial training, strategies used to help patients integrate self-care into their daily lives, and self-managed PA efficacy. Tables were produced for every oncological side effect that BCS might experience: lymphedema, arthralgia, cancer-related fatigue, a decline in physical parameters, treatment-related cardiotoxicity, peripheral neurotoxicity, and a possible decline in the quality of life.

**Conclusions:**

Self-managed PA has the potential to improve BCS oncological issues. Professionals can adopt many strategies to support patients and empower them with long-lasting self-care competencies. This scoping review provided a comprehensive and easy-to-consult overview of self-managed PA interventions for BCS. We also provided recommendations for future primary studies and secondary synthesis.

## Introduction

Cancer has one of the highest incidences among diseases in developed countries. Breast cancer especially, is the leading female cancer, representing 15% of all new tumor diagnosis every year [1].

As of 2016, preventive screening programs and increasingly precise therapies have led to enormous progress in terms of survival rates for approximately 90% of women, five years after being diagnosed [1]. However, breast cancer survivors (BCS) experience a range of long-term physical problems caused by the disease itself and, very frequently, by the therapies they have received [2]. The main consequences are upper limb lymphedema following lymph node dissection (total or partial—sentinel lymph node only) and/or radiotherapy, arthralgia caused by aromatase inhibitors, cancer-related fatigue (CRF), reduced bone mineral density, decline of muscle and body structure, cardiotoxicity, and peripheral neurotoxicity [2]. Consequently, these issues affect patients’ quality of life (QoL), starting a dangerous vicious cycle between the above outcomes and participation in social, work, and family contexts [3].

Given the chronicity of most oncological outcomes even years after treatment, interventions to reduce or control their development are necessary. Several strategies in current literature are described as being effective in controlling oncological symptoms, including physical exercise, psychological techniques such as cognitive behavioral therapy, pharmacological interventions, or nutritional therapies [2]. Within a multi-disciplinary and patient-centered rehabilitative approach, the intervention of a physiotherapist or, more generally, of an exercise expert plays a fundamental role for the well-being and recovery of the patient [4].

Standard physiotherapy and physical activity (PA) programs offered to BCS often take place while their therapies are still in progress but usually have a limited duration in time, ending upon discharge from the hospital inpatient regime or shortly thereafter [5]. In the long run survivors are left to fend for themselves, without proper training in order to have a real impact in their lifestyles [5]. In fact, less than 30% of BCS have an adequate level of weekly PA, and some studies report that more than 80% of them have not been properly educated about the benefits of exercise during, or after their treatment [6].

This practice is at odds with the Chronic Care Model designed by Wagner in 1998 [7] and later updated by Barr and colleagues in 2003 [8], in which the concept of self-care support was one of the main points. Thanks to this practice, patients were progressively guided towards learning how to manage their health, promote self-empowerment, and become capable of continuing their care independently in the long term, thus preventing symptom relapse [7]. Literature is promoting rehabilitation programs where patients with chronic diseases become semi-autonomous in the management of their symptoms by simply continuing the activities previously acquired from their healthcare professionals, and recent systematic reviews support the referral to Wagner’s model for organizing these projects [9]. Moreover, additional elements can hinder these long-lasting supervised programs, such as financial issues, the distance travelled to and from the facilities, and the availability of caregivers to accompany BCS during their treatments [10]. These elements further support the need for integrated self-managed rehabilitation programs after cancer therapies. Since different cancer outcomes affect women in several personal spheres, in this study we have focused not only on assessing each single outcome but also on investigating how the latter impacts women’s QoL and social participation from a biopsychosocial perspective as described by the International Classification of Functioning, Disability and Health [11, 12].

Since the topic of self-managed PAs for BCS is extensive and it has never been studied jointly, a scoping review seemed the way forward to investigate it. Scoping reviews are a way to examine and map existing literature on a wide-ranging topic, even when available evidence is still limited or is still in the initial stages, and to highlight the knowledge gaps that need to be filled [13, 14]. Unlike systematic reviews, scoping reviews do not mean to establish the efficacy of a treatment or to produce a critically appraised and synthesized answer to a specific question [15].

We conducted a scoping review to map available literature on how self-managed PA is delivered and how it affects oncological outcomes in BCS.

## Methods

We followed the guidelines specific for scoping reviews [13], which are the extension of the PRISMA guidelines for systematic reviews [16]. The methodological process includes six stages, outlined below.

### Stage 1: Identifying the research question

The leading questions of our investigation were: I) Which types of self-managed PAs are used in literature to treat cancer-related outcomes in BCS? II) How are they administered, and what is the frequency and the dosage proposed to counteract the symptoms? III) What effects are obtained on outcome? IV) What strategies are used to help patients embrace self-care in their daily routine?

### Stage 2: Identifying relevant studies

Systematic searches were designed, refined, and conducted between February 2021 and April 2022. No time limitations were set, and the following databases were investigated: Medline, CINAHL, and Cochrane. Only articles published in English were taken into consideration.

Keywords searched included “tumor”, “malignancy”, “cancer survivors”, “self-care”, “self-cure”, “self-management”, “physical therapy modalities”, “rehabilitation”, “physiotherapy”, “exercise”, and “home-based care”. Medical Subjects Headings (MeSH) were used, when available, to ensure consistency of the search terms (full search strategies can be found in Table 1). Additional papers not intercepted by the search string were added later by handsearching the reference lists of the identified studies.

**Table 1:** Search strategies.

Database	Strategy
**MEDLINE (PubMed)**	("Breast Neoplasms"[Mesh] OR breast cancer OR breast carcinoma OR mammary cancer OR breast tumor OR breast tumour OR breast neoplasm) AND ("Self Care"[Mesh] OR self cure OR self care OR self management OR home-based OR unsupervised) AND ("Physical Therapy Modalities"[Mesh] OR rehabilitation OR physical therap* OR physiotherapy OR exercise* OR physical activity)
**Cochrane Database**	(MeSH descriptor: [Breast Neoplasms] explode all trees OR breast cancer OR breast carcinoma OR mammary cancer OR breast tumor OR breast tumour OR breast neoplasm) AND (MeSH descriptor: [Self Care] explode all trees OR self cure OR self care OR self management OR home-based OR unsupervised) AND (MeSH descriptor: [Physical Therapy Modalities] explode all trees OR rehabilitation OR physical therap* OR physiotherapy OR exercise* OR physical activity)
**Cinahl Database**	(MH "Breast Neoplasms" OR breast cancer OR breast carcinoma OR mammary cancer OR breast tumor OR breast tumour OR breast neoplasm) AND (MH "Self Care" OR self cure OR self care OR self management OR home-based OR unsupervised) AND (MH "Physical Therapy" OR rehabilitation OR physical therap* OR physiotherapy OR exercise* OR physical activity)

### Stage 3: Selecting studies

Two reviewers (MCBO and AM) screened all articles and assessed them according to the eligibility criteria. As clarified by the scoping review guidelines [13, 14, 16], no quality appraisal of the included studies was performed. Any discrepancy was solved by involving a third reviewer (IC). If the full text was not available, authors were contacted via email, and the studies were excluded only if authors did not reply. The eligibility criteria are presented following the PICO framework:

#### Types of studies

We included primary quantitative literature as experimental or observational studies that involved self-managed PA after a specific prescription. Initial recommendations by a professional were necessary for the inclusion. Study designs such as protocols, intervention development, case-control, or qualitative studies, and secondary literature such as reviews and meta-analysis were excluded. We also excluded literature in which full text was not available, since these could not provide enough details about the variables we investigated. Finally, studies that were just pilot studies of trials already included were also discarded, as running the risk of overestimating the results.

#### Population

Adult BCS were the population of interest for this review. We included BCS who had at least started their cancer therapies and were at any stage of the disease that implied a potential need for recovery from long-term outcomes affecting women. We excluded BCS whose cancer stage required only palliative interventions. Studies involving minors or adults recovering from pediatric tumors were excluded.

#### Intervention

We considered self-managed any PA program that initially started under clinical prescription and later were performed autonomously. Patients could be supervised and trained by a professional in the initial stages, then the program had to involve at least a one-month self-managed activity before evaluating the patient again at the follow-up. During the self-care phase, professionals could offer in-person support meetings to increase patient motivation or go over the exercise, but these could not exceed a frequency of less than a fortnight. No frequency limits were set for remote support, such as phone calls or texting. Interventions could involve multidisciplinary approaches, combining PA as the primary intervention along with diet or psychological therapies.

#### Comparison

Since we included both single-arm and multiple-arm trials, the interventions could be compared with any type of control.

#### Outcome

Outcome measures had to be related with the long-term issues experienced by BCS: lymphedema, arthralgia, CRF, reduced bone mineral density, decline of muscle and body structure, cardiotoxicity, and peripheral neurotoxicity. We also included outcome measures for patient’s QoL.

#### Context

No limitations were set regarding the context of the studies.

### Stage 4: Charting the data

Relevant data were extracted from each study and grouped in tables. When in doubt we reviewed changes in the table headings to increase accuracy.

### Stage 5: Summarizing and reporting the data

Information on the included control groups, type of self-care PA, recommended frequency, duration of interventions, and the professional leading the program were extracted from each included study and summarized in appropriate tables. Details of the strategies used by researchers to guide BCS toward autonomy were also collected: supervision at the beginning of treatment and techniques of ongoing support during self-care were categorized and collected in another table. A narrative synthesis of the most relevant topics was provided as well. Further characteristics of each study are available in the S1 Table.

### Stage 6: Consultation

One of the authors (MBL), expert in the field of oncological Physical and Rehabilitation Medicine, was consulted during the whole review process to help us set inclusion criteria and to identify important literature we could have inadvertently missed. Findings were discussed again at the end of the writing, to increase accuracy on key points.

## Results

**Fig 1:**
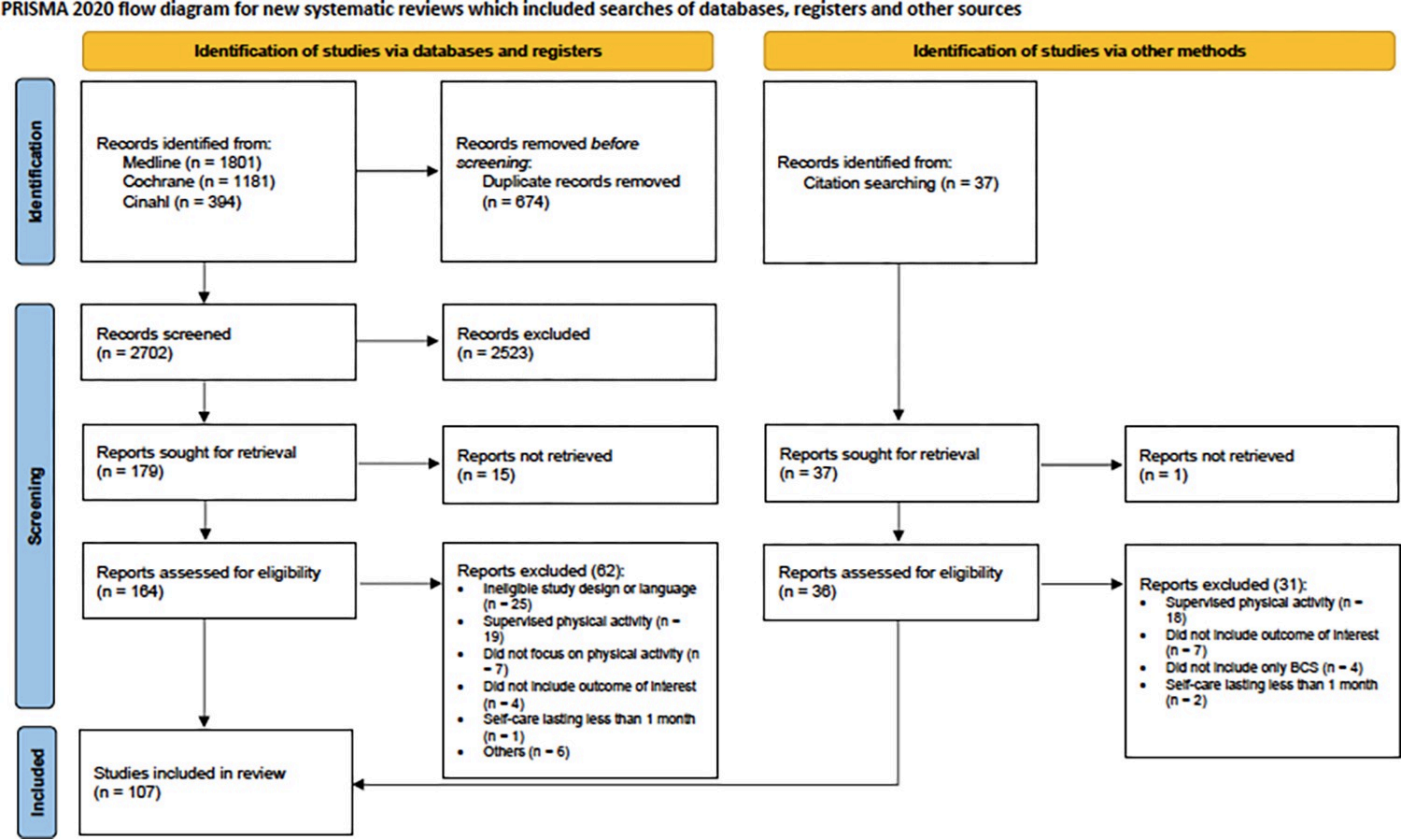
Flow chart of the search conducted.

The literature search led to 3,376 records. We screened the titles and abstracts for a total of 2,702 non-duplicate studies, and we excluded 2,522 according to the eligibility criteria, mainly because of inappropriate study designs or the lack of focus on BCS. One hundred-eighty studies were selected for full-text screening and 102 were finally included. Five additional studies were retrieved by handsearching, leading to a total of 107 studies. However, one study described two separate different trials [17] and was therefore considered twice, leading to 108 included studies. Fig 1 summarizes the flow of the search process corresponding to PRISMA guidelines for scoping reviews [13, 16]. Details about each individual study are available in the S1 Table.

Out of the 108 studies, 64% (69/108) were randomized controlled trials (RCT) with two/three/four arms; 7% (8/108) were pilot studies; 24% (26/108) had a single-arm design, and 5% (5/108) had other study designs. Eighty-two studies compared the self-care intervention to at least one control group, as illustrated in Table 2. Included samples among studies ranged from 6 to 692 BCS.

**Table 2:** Intervention delivered or activity performed by the control group in the studies included in this review.

Type of control group	Number of studies
*Two-arm studies (n = 77)*	
Active control group	12
Similar activity with supervision	5
Diet	3
Educational/psychological control group	2
Usual care	55
*Three/four-arm studies (n = 5)*	
Active control group and usual care	3
Educational/psychological control group and usual care	1
Similar activity with supervision and usual care	1
*Without control group (n = 26)*	

Details of the self-care PA prescribed to BCS are presented in Table 3. These mainly involved aerobic exercise alone, combined exercise programs (including aerobic, resistance, flexibility, and balance exercises), and multidimensional programs (combining PA with nutritional or psychological interventions). Complex decongestive therapy and upper limb exercises were specific interventions for treating lymphedema. Finally, a minority of studies offered BCS mind-body practices such as Yoga, Tai Chi, or Qigong, or the performance of resistance exercises only. The duration of the studies was varied considerably, ranging from 1–2 months to over a year in a few cases. A more homogeneous consensus surrounded the recommended frequency: 73% (79/108) suggested PA at least three times a week, or even daily, and only 10% (11/108) suggested a lesser frequency. On the other hand, 17% (18/108) of the studies did not specify how frequently a patient should exercise.

**Table 3:** Characteristics of self-managed interventions, reported in terms of type of activity, duration, recommended frequency, and professional managing the program.

Characteristics of the interventions	Number of interventions (n = 108)
*Type of activity*	
Aerobic exercise program	39
Resistance exercise program	6
Combined exercise program (aerobic, resistance, stretching, flexibility)	25
Mind-body practice (Yoga, Tai Chi, Qigong)	11
Multidimensional program (physical, nutritional, psychological)	12
Complex decongestive therapy	12
Upper limb exercise program	3
*Duration*	
1–2 months	30
3–5 months	31
6–11 months	32
≥ 1 year	15
*Frequency*	
1–2 times / week	11
≥ 3 times / week	57
Daily	22
Not indicated	18
*Responsible professional*	
Exercise expert	25
Physiotherapist	21
Multidisciplinary team	12
Nurse	11
Mind-body teacher	8
Others	2
Not specified	29

Regarding the person in charge of the intervention, the most frequent professional involved in the self-management of BCS were exercise experts and physiotherapists. Eleven percent (12/108) of the studies involved a multidisciplinary team, with at least two different professionals working together.

Regarding the strategies used by professionals to help patients integrate self-care activities and behaviors into their daily lives, we differentiated between interventions delivered at the beginning of the program targeting autonomy and those adopted during the entire self-care period (see Table 4). A large proportion of researchers–around 40% (43/108)—took advantage of an initial period of supervision (from ten days to one year), including the hospitalization period, to properly train and monitor patients. Other studies provided a single consultation with a practical demonstration during the first meeting. During the self-care phase, most of the studies verified the patient’s self-care status via phone calls or texting, while 16% (17/108) were scheduled meetings with the professionals or sporadic supervised sessions to revise the program jointly. However, 20% (22/108) did not provide any initial training and 39% (42/108) did not provide any ongoing support.

**Table 4:** Supervision provided and strategies adopted to promote self-care behaviors in breast cancer survivors’ routine.

Intervention	Number of studies (n = 108)
*Initial supervision*	
Single initial consultation	40
Frequent contacts during the initial period	3
Intensive training during the initial period	43
Not delivered	22
*Strategies adopted during self-care*	
Periodic calls or texting	46
Regular meetings	16
Sporadic supervised sessions	1
Educational support	3
None	42

The following paragraphs summarize the main outcome measures and efficacy results as reported by the included studies, separating each outcome BCS could experience after an oncological therapy. We recommend caution when interpreting these results, as this scoping review only provides a descriptive synthesis, without statistical analysis.

### Lymphedema

Twenty-seven studies explored the efficacy of self-care PA in reducing lymphedema [18–44].

The main measures employed to evaluate lymphedema were the computation of interlimb volume differences (as measured by volume, tape circumference calculation, or perometry) and bio-impedance spectroscopy. Additional measures implemented to get a complete view of the arm status were the measurement of shoulder range of motion and of pain assessment. Two out of eleven RCTs reported self-care to be effective in reducing lymphedema in BCS compared to usual care, especially when complex decongestive therapy was performed. In three out of eleven RCTs lymphedema was reduced when compared to baseline values, however, self-care was comparable to the activities performed by the control groups.

### Arthralgia

Only three studies recorded the effects of self-care PA on arthralgia and one of them was an RCT [45–47].

Outcome measures used specific scales for joint pain, as the Arthritis Impact Measurement Scale and the Western Ontario and McMaster University Osteoarthritis Index. In the long run, significant differences pertaining to the arthralgia domain were found for all three studies, nonetheless, no study demonstrated the superiority of self-care over usual care.

### Cancer-related fatigue

Fifty studies explored the efficacy of self-care PA in reducing CRF in BCS [46, 48–96].

About 15 different scales have been used across studies to measure the perception of CRF and its impact on patients’ daily life. Among these, the Functional Assessment of Cancer Therapy–Fatigue and the Piper Fatigue Scale were mostly employed. Researchers then often combined functional tests to assess patients’ fatigue such as the 6-Minute Walking Test (6MWT) or peak oxygen uptake (VO2) computation. Thirty-nine percent (15/38) of the RCTs claimed self-managed PA to be effective in reducing fatigue compared to other activities. Other seven RCTs found improvements in CRF in the long run, even though it did not record between-group differences. At the end of the interventions, the sixteen remaining RCTs did not register any changes in the symptoms.

### Physical parameters

A total of 37 studies investigated the efficacy of self-care PA in controlling body composition and loss of bone or muscle tissues [18, 24, 38, 45, 52, 54, 55, 65, 68, 69, 71, 76, 81, 83, 86, 90, 96–115].

The body mass index was the main measure for assessing body composition, with particular focus on lean and fat mass. Bone mineral density was usually computed by dual-energy X-ray absorptiometry. Muscle strength was evaluated with functional tests depending on the muscles analyzed, as with a dynamometer or by maximum repetition number. Results on effectiveness were heterogeneous among RCTs: 4/17 found self-care to be effective in controlling weight gain and body mass index compared to control groups; 0/6 reported significant results on bone mineral density when comparing the interventions and control groups, and 5/14 registered an increase in muscle strength, enough to be statistically different when compared to that of the control group.

### Cardiotoxicity

Cardiotoxicity leads to reduced cardiac function and potential fatal adverse events. The effects of self-care PA can be indirectly calculated by assessing cardiovascular fitness. A total of 20 studies evaluated this parameter [38, 52, 54, 55, 65, 68, 69, 81, 83, 85, 86, 92, 95, 97, 101, 106, 108, 110, 111, 116].

The main measures used to assess cardiovascular fitness were the VO2 computation, the distance covered during the 6MWT, and the heart rate under stress conditions. Four out of 14 RCTs found greater positive effects on cardiovascular fitness at the end of the self-managed programs compared to control groups. Seven further RCTs registered significant improvements in VO2 Maximum Test over time but not in the between groups.

### Peripheral neurotoxicity

Two studies investigated the efficacy of self-care PA in controlling neuropathic symptoms in BCS and one of them was an RCT [117, 118].

Both used specific validated scales: the Neuropathic Pain Scale for Postsurgical Patients and the Leeds Assessment of Neuropathic Symptoms and Signs. Both studies recorded significant differences compared with pre-interventions values.

### Quality of life

Fifty-five studies also investigated changes in QoL following self-care PA programs [17, 20, 25, 26, 30, 32, 33, 39, 41, 44, 45, 47–49, 52–55, 61–63, 65, 66, 69, 77, 81–83, 86, 90–95, 98, 100–103, 105, 106, 108, 111, 113–116, 118–124]. The outcome measures mostly used were the European Organization for the Research and Treatment of Cancer Quality of Life Questionnaire, the Functional Assessment of Cancer Therapy–Breast, and the Short Form Health Survey 36. Twenty-five percent (9/36) of the RCTs recorded significant changes in QoL after self-care compared to the control groups’ activities. Forty-four percent (16/36) of studies recorded improvements over time and the remaining thirty-one percent (11/36) found self-care to be ineffective in regaining QoL.

A summary of the results of the RCTs with respect to the initial supervision delivered and the strategies employed to help patients embrace self-care is provided in Table 5. When a study evaluated more than one outcome, this was considered multiple times.

**Table 5:** Treatment efficacy (count), considering only RCTs.

Study results	Results counting
***Initial supervision***	
*Single initial consultation*	
Effective treatment *^,^^†^	11
Effective treatment ^†^	18
Ineffective treatment	14
*Frequent contacts during the initial period*	
Effective treatment *^,^^†^	1
Effective treatment ^†^	1
*Intensive training during the initial period*	
Effective treatment *^,^^†^	15
Effective treatment ^†^	19
Ineffective treatment	23
*Not delivered*	
Effective treatment *^,^^†^	9
Effective treatment ^†^	6
Ineffective treatment	9
***Strategy to increase adherence***	
*Periodic calls or texting*	
Effective treatment *^,^^†^	20
Effective treatment ^†^	20
Ineffective treatment	17
*Educational or support groups*	
Effective treatment *^,^^†^	1
Ineffective treatment	3
*Regular visits*	
Effective treatment *^,^^†^	6
Effective treatment ^†^	11
Ineffective treatment	7
*Sporadic supervised sessions*	
Effective treatment *^,^^†^	1
Effective treatment ^†^	1
*None*	
Effective treatment *^,^^†^	8
Effective treatment ^†^	12
Ineffective treatment	19

* Statistically significant between-groups difference (compared to the control group): the effect of smPA is greater than that of the control treatment

† statistically significant within-group difference in the intervention group (compared to baseline values): the effect of smPA is equal to that of the control treatment. a Frequent contact during the initial period included studies where authors repeatedly checked for patients’ adherence and comfort after the prescribed exercises via weekly in-person meetings, phone calls and emails. b Intensive training during the initial period involved studies in which authors provided a supervised period at the beginning of the program in order to educate patients and improve their long-term adherence. Note: each study was considered for each variable analyzed

## Discussion

In oncology, the topic of self-care is an increasingly heartfelt topic, as demonstrated by the high number of studies published in the last decade. Cancer survivors experience a wide range of issues starting with the diagnosis and continuing even several years after the end of a treatment [2, 125]. In the long term these patients could benefit from new ways of managing their symptoms so as not to be dependent on health professionals. To the best of our knowledge, this is the first study mapping available literature on self-managed PA used to control long-term issues experienced by BCS.

Our review included both BCS patients still undergoing adjuvant therapies and those who had already finished cancer treatments. The goals set for these samples were different: for the former, the target of rehabilitation was to prevent and contain the onset of symptoms; for the latter, it was to reduce them. In line with the scoping review’s aims, we did not mean to draw efficacy results. Therefore, this heterogeneity does not produce a bias in our results. On the contrary, we considered it a positive sign that several authors suggested a self-managed PA program when active cancer treatments were still in place, reflecting a stepwise approach needed for this population. In fact, although the purpose of PA changes according to the various stages of treatment, a step-by-step educational support can be implemented from the very beginning. This will undoubtedly help BCS to adopt and maintain a healthier lifestyle in the years to come.

Among the 108 studies that met the inclusion criteria for this scoping review, we found many different types of programs for self-managed PA (see Table 3). Most of the programs delivered involved aerobic exercise or several activities combined–e.g., a mix of aerobic, resistance, flexibility, and balance exercises. New multidimensional approaches—e.g., including mind-body practices—are also on the rise, as seen in 21% (23/108) of our findings. The availability of such a broad selection of self-care activities that can be selected and combined in tailor-made programs, could encourage treatment adherence, and possibly determine long-lasting results [126–128].

In this review, treatment dosage was considered in terms of both duration of the self-managed activity and its recommended frequency. The duration of these programs varied somewhat among studies, ranging from 1 to 24 months (see Table 3). On the one hand, this lack of unanimity might depend on the complexity and the differences among oncological issues. On the other hand, the very short intervention duration (1 month) selected in some studies could have been chosen to ensure study feasibility. Future studies ought to investigate what the minimum duration is, in order to achieve clinically meaningful results, along with the ensuing long-term side effects. Furthermore, when reporting the results of new trials, authors should explain the reasons underlying the length of the intervention period chosen.

Almost all studies agreed on relatively frequent PA throughout the week, mainly suggesting self-care practices at least three times per week. This is in line with PA guidelines for cancer survivors that recommend 150 min/week of aerobic exercise and 2–3 weekly sessions of resistance activity [129–131]. Moreover, it is consistent with the need to create a continuum in the care that becomes part of the patients’ daily routine [7]. It is worth mentioning that as many as 18 included studies did not specify with which frequency BCS should be practicing. Since this limitation prevents the replicability of the studies, and does not allow to properly analyze their results in systematic reviews, the information on the recommended amount of weekly activity should always be reported in future studies.

Several healthcare professionals prescribed self-care programs. This appeared to be a relevant element for an in-depth analysis of the interventions. We therefore decided to include it among the variables retrieved from each study (see the S1 Table). As many as nine different professionals, both from healthcare and other non-scientific backgrounds, oversaw the PA programs, as shown in Table 3. In our opinion, this variability among professionals highlights the possibility of creating multidisciplinary teams supporting women, favoring a gradual transition from the hospitalization phase to that of a home routine [132–137]. Health professionals can easily handle BCS during the post-surgical acute phase and the follow-ups, while different professionals could follow patients during daily long-term management, in a suitable environment (support group activities, psychological and nutritional support groups, adapted PA groups, mind-body activities performed in gyms and sport facilities), according to individual needs and preferences and with no further burden to the national health systems.

Different levels of self-care were the main focus of this review: from complete patient autonomy to hybrid conditions in which strategies of ongoing patient support were used. In most studies, more complex interventions included strengthening exercises, mind-body techniques—e.g., Yoga—as well as self-managed lymphatic drainage and bandaging that could be more complex to put into practice independently. In the remaining studies, aerobic exercise consisted of simply reaching a set amount of weekly walking and was self-managed. This difference justifies the initial presence and supervision by professionals, as stated in most of the included studies (see Table 3). Here, the practitioner, not only checked that the exercises were being carried out correctly and corrected the patients, even adapting the program as they went along, but also acted as a bridge to gradually prepare the patients to the importance of independent PA. This is in line with the Chronic Care Model [7]. After the initial training period, contact with professionals during the self-care phase, albeit sporadic follow-ups, was to help patients not feel abandoned once their therapies were over, developing self-empowerment and self-efficacy. Moreover, literature specific to the BCS population reports that women may be at risk of neglecting their health because of the burden related to the family management [128]. A periodic reminder about the importance of ongoing self-care could help them re-establish their priorities, set short-term achievable goals, and stay active in the years to come.

In addition to this continuous support during the chronic stage of the disease, a new field of oncological rehabilitation pertaining to pre-habilitation is gaining ground—i.e., promoting health-optimizing behavior soon after the diagnosis so as to mitigate the unwanted consequences of future cancer treatments [138]. Cancer diagnosis has been proven to be a teaching experience that results in health and behavioral changes [139]. For this reason, pre-habilitation could further support BCS in a gradual understanding of the importance of self-care in the years to come. Since no study addressing this topic was found for BCS, future studies should explore this opportunity further.

### Implications for the future

In line with the aim of this scoping review, we have mapped the recommendations for self-care through physical activity available in literature. We also collected the outcomes used in literature to assess several health issues that women might experience (see the S1 Table). These can be used by researchers when designing new trials. The ratio of effective and non-effective treatments reported in Table 5 is a mere descriptive analysis of the results in literature. A statistically-based synthesis to determine whether a treatment is effective goes beyond the aim of this scoping review–i.e., a meta-analysis would be appropriate, where effect size is computed and weighted based on sample sizes and average. In this paper we intentionally did not perform a quantitative meta-analysis synthesis: a systematic review would have required summarizing all the information in a single effectiveness index. This would have led to the loss of the specific characteristics of each individual study—e.g., considering aerobic exercise on par with yoga, or programs involving an initial supervised period on par with those self-managed from the very beginning–to favor concise and homogeneous results. Moreover, the heterogeneity of our included studies in terms of stages of breast cancer, type of intervention, and outcome measure would have prevented us from performing a single large meta-analysis. The current scoping review provides a detailed overview on the topic by identifying areas of interest and the confounding factors that future clinical trials and meta-analyses should consider. For future systematic reviews, we suggest a careful selection of the studies by identifying smaller homogeneous subgroups with similar parameters—e.g., similar treatments, comparable levels of self-care support and follow-up, grouping samples according to the cancer stages—to obtain more accurate recommendations when choosing the best self-managed interventions.

Regarding future new clinical studies, we recommend that authors state clearly all treatment variables as shown in Tables 3 and 4 - e.g., weekly frequency, professional in charge, strategies used to help patients move towards autonomy—as these could constitute factors influencing treatment efficacy. Most of the studies available in literature compared self-care interventions with usual care groups, and women only received “How To” instructions mainly with pamphlets (see Table 2). Comparing self-care programs to similar supervised programs would undoubtedly be beneficial for future research, given the already widely demonstrated effectiveness of the latter [3, 4, 126, 140, 141]. Knowing whether the same results can be obtained both independently and with constant supervision might help clinicians design tailored treatments according to the needs and circumstances of each individual patient—e.g., distance from the hospital.

When considering future systematic reviews, we suggest conducting a synthesis of the effectiveness of the interventions by investigating: 1) which type of self-managed PA is more effective based on each outcome; 2) the minimal duration to have a positive effect, and 3) whether the choice of initial training and ongoing support from professionals promotes better outcomes compared to complete self-care. Once this is established, this would allow national health systems to set up evidence-based and detailed self-care programs for BCS in an effort to reduce re-hospitalizations and new medical interventions due to exacerbation of symptoms that have not been adequately controlled [142]. Monitoring health care spending and simultaneously improving patients’ lives are the goals to strive for in this new era of chronic cancer management.

Finally, health professionals can also benefit from this review during their daily clinical practice. Indeed, clinicians may need to look up the best solutions and guidelines when dealing with BCS and their multifaceted long-term issues and, unfortunately, they are not always familiar with database research. In this scoping review, professionals can immediately find a comprehensive and user-friendly summary of the available literature on self-managed PA programs.

### Strengths and limitations

This is the first scoping review that maps existing primary literature on self-managed PA programs for BCS in terms of types of exercise, duration, frequency, professionals, and addressed health issues. The main strengths of our study are a robust search string developed with a specialist from our Institution (MCBA) who researched the main databases. Both entirely self-managed programs, and programs that included strategies for patient guidance and support were included. Moreover, we considered eligible both traditional PA such as aerobic and resistance exercise, and new types of practices such as Yoga, Tai Chi, Qigong, or multidimensional programs. This allowed for a broader and more comprehensive study of available evidence.

The main limitation of the current review is the risk of not having retrieved all the studies on self-care. This could be due to the novelty in this cancer rehabilitation field since some studies might not yet be labeled correctly in the databases. To reduce these odds, we performed a thorough hand-search starting from the bibliography of the studies found with the electronic search. In addition, this review did not cover all types of self-care for BCS, but only those that involved PA as the primary intervention. Programs primarily based on psychological or nutritional interventions were not included and should be considered in separate reviews, given the very different rationale of the treatments. Finally, with our team’s experience and with help from experts in the field we were able to highlight the main issues common to cancer therapies. However, we may have skipped some important domains. Pain, for example, is a relevant side effect that BCS experience. We chose to consider it as a complex multifactorial condition integrated within other issues, such as lymphedema, arthralgia, and neurotoxicity (as can be seen in the S1 Table), and to therefore not analyze it as a separate domain.

## Conclusions

BCS experience several health issues following cancer therapies and may benefit from different types of physical activity in order to manage them. We have mapped the evidence surrounding exercise-based interventions when these are self-managed by women after being prescribed by a doctor. Professionals working with BCS have several strategies at their disposal to support patients by empowering them with long-lasting self-care skills. Recommendations for clinical studies and systematic reviews were provided, along with suggestions about the grey areas still to cover and develop.

## Supporting information

S1 Checklist PRISMA checklist. (DOCX)

S1 Table List of all studies included in the scoping review and their main characteristics. * statistically significant between-groups difference (compared to the control group): the effect of self-managed PA is greater than that of the control treatment; † statistically significant within-group difference in the intervention group (compared to baseline values): the effect of self-managed PA is equal to that of the control treatment.
(DOCX)

## List of abbreviations

BCS: Breast Cancer Survivors
CRF: Cancer-Related Fatigue
PA: Physical Activity
QoL: Quality of Life
RCT: Randomized Controlled Trial
VO2: peak Oxygen uptake
6MWT: 6-Minute Walking Test
