# Supplementary material for: Self-managed physical activity in breast cancer survivors: A scoping review
Source: PLoS One. 2023 Apr 24;18(4):e0284807. doi: 10.1371/journal.pone.0284807 (PMC10124851; doi:10.1371/journal.pone.0284807)
Supplement: S1 Table — * statistically significant between-groups difference (compared to the control group): the effect of self-managed PA is greater than that of the control treatment; † statistically significant within-group difference in the intervention group (compared to baseline values): the effect of self-managed PA is equal to that of the control treatment. (DOCX) [file pone.0284807.s002.docx]

**S1 Table. List of all studies included in the scoping review and their main characteristics.**

| **Study and year** | **Outcome** | **Study design** | **Sample size** | **Health professional leading the intervention** | **Intervention** | **Strategy to maintain adherence** | **Recommended frequency** | **Duration** | **Initial supervision** | **Control group** | **Outcome measures** | **Study results** |
| --- | --- | --- | --- | --- | --- | --- | --- | --- | --- | --- | --- | --- |
| Ammitzbøll et al. 2019 | Lymphedema | RCT | 158 | Physiotherapist | Resistance exercise program | Regular visits | ≥ 3 times/week | 6-11 months | Intensive training during the initial period | Usual care | Interlimb volume difference, Interlimb mass difference, NRS | Ineffective treatment |
|  | Bone, muscle, BMI | RCT | 158 | Physiotherapist | Resistance exercise program | Regular visits | ≥ 3 times/week | 6-11 months | Intensive training during the initial period | Usual care | 7-repetition maximum tests (shoulder abduction, elbow flexion/extension, leg press), handheld dynamometry | Effective treatment†* |
| Ammitzbøll et al. 2019 (2) | QoL | RCT | 158 | Physiotherapist | Resistance exercise program | Regular visits | ≥ 3 times/week | 6-11 months | Intensive training during the initial period | Usual care | EORTC-QLQ-C30 (also pain domain), FACIT-F | Effective treatment† |
| Ammitzbøll et al. 2020 | Neurotoxicity | RCT | 158 | Physiotherapist | Resistance exercise program | Regular visits | ≥ 3 times/week | 6-11 months | Intensive training during the initial period | Usual care | Neuropathic Pain scale for Postsurgical patients | Effective treatment† |
| Andersen et al. 2000 | Lymphedema | RCT | 42 | Physiotherapist | Complex decongestive therapy | None | Not specified | ≥ 1 year | Intensive training during the initial period | Active group | Arm circumferences, volumetry, symptoms questionnaire | Ineffective treatment |
| Arinaga et al. 2019 | Lymphedema | Pilot study | 43 | Not specified | Complex decongestive therapy | None | Daily | 6-11 months | Not delivered | Usual care | L-Dex, interlimb volume difference, LYMQOL | Ineffective treatment |
|  | QoL | Pilot study | 43 | Not specified | Complex decongestive therapy | None | Daily | 6-11 months | Not delivered | Usual care | SF-8 | Ineffective treatment |
| Baruth et al. 2015 | CRF | Pilot study | 32 | Others | Aerobic exercise program | Periodic calls | ≥ 3 times/week | 3-5 months | Single initial consultation | Usual care | FACT-F, CHAMPS | Effective treatment†* |
|  | QoL | Pilot study | 32 | Others | Aerobic exercise program | Periodic calls | ≥ 3 times/week | 3-5 months | Single initial consultation | Usual care | SF-36, International Breast Cancer Study Group QOL Core Questionnaire | Effective treatment†* |
| Basen-Engquist et al. 2020 | QoL | RCT | 38 | Not specified | Combined exercise program (aerobic, resistance, flexbility, stretching, balance) | Regular visits | ≥ 3 times/week | 1-2 months | Intensive training during the initial period | Usual care | SF-36 | Ineffective treatment |
| Baumann et al. 2017 | CRF | RCT | 194 | Physiotherapist | Aerobic exercise program | Sporadic supervised sessions | Not specified | ≥ 1 year | Intensive training during the initial period | Usual care | MFI | Effective treatment†* |
|  | QoL | RCT | 194 | Physiotherapist | Aerobic exercise program | Sporadic supervised sessions | Not specified | ≥ 1 year | Intensive training during the initial period | Usual care | EORTC-QLQ-C30 | Effective treatment† |
| Brown et al. 2015 | Lymphedema | Observational study | 128 | Multidisciplinary team | Resistance exercise program | Periodic calls | 1-2 times/week | 6-11 months | Intensive training during the initial period | Not included | Volumetry, arm circumferences, bio-impedance spectroscopy, Norman lymphedema survey (also pain domain) | Ineffective treatment |
| Cadmus et al. 2009 (a) | QoL | RCT | 50 | Not specified | Aerobic exercise program | Periodic calls | ≥ 3 times/week | 6-11 months | Single initial consultation | Usual care | FACT-B, SF-36 | Effective treatment† |
| Cadmus et al. 2009 (b) | QoL | RCT | 75 | Not specified | Aerobic exercise program | Periodic calls | ≥ 3 times/week | 6-11 months | Intensive training during the initial period | Usual care | FACT-B, SF-36 | Effective treatment† |
| Cansız et al. 2022 | Lymphedema | Single-arm trial | 44 | Nurse | Complex decongestive therapy | Periodic calls | ≥ 3 times/week | ≥ 1 year | Single initial consultation | Not included | Arm circumferences, volumetry, DASH (also pain domain) | Effective treatment† |
| Carayol et al. 2019 | CRF | RCT | 143 | Exercise expert | Multidimensional program (physical, nutritional, physchological components) | Regular visits | ≥ 3 times/week | ≥ 1 year | Single initial consultation | Usual care | MFI | Effective treatment†* |
|  | Bone, muscle, BMI | RCT | 143 | Exercise expert | Multidimensional program (physical, nutritional, physchological components) | Regular visits | ≥ 3 times/week | ≥ 1 year | Single initial consultation | Usual care | BMI, strength | Effective treatment† |
|  | QoL | RCT | 143 | Exercise expert | Multidimensional program (physical, nutritional, physchological components) | Regular visits | ≥ 3 times/week | ≥ 1 year | Single initial consultation | Usual care | EORT-QLQ-C30 | Effective treatment†* |
| Carter et al. 2016 | CRF | RCT | 152 | Exercise expert | Aerobic exercise program | Regular visits | ≥ 3 times/week | 1-2 months | Intensive training during the initial period | Usual care | 5-point linear scale, VO2 | Effective treatment†* |
| Carter et al. 2018 | Bone, muscle, BMI | RCT | 27 | Exercise expert | Aerobic exercise program | Regular visits | ≥ 3 times/week | 1-2 months | Intensive training during the initial period | Usual care | BMI | Effective treatment† |
|  | Cardiotoxicity | RCT | 27 | Exercise expert | Aerobic exercise program | Regular visits | ≥ 3 times/week | 1-2 months | Intensive training during the initial period | Usual care | VO2 maximum test | Effective treatment† |
| Chandwani et al. 2014 | CRF | Three-arm trial | 163 | Mind-body teacher | Mind-body practice (yoga, qigong, tai chi) | None | Not specified | 1-2 months | Intensive training during the initial period | Active group / Usual care | BFI | Effective treatment† |
|  | QoL | Three-arm trial | 163 | Mind-body teacher | Mind-body practice (yoga, qigong, tai chi) | None | Not specified | 1-2 months | Intensive training during the initial period | Active group / Usual care | SF-36 | Effective treatment†* |
| Chaoul et al. 2018 | CRF | Three-arm trial | 227 | Multidisciplinary team | Mind-body practice (yoga, qigong, tai chi) | Regular visits | Daily | ≥ 1 year | Intensive training during the initial period | Active group / Usual care | BFI | Effective treatment† |
| Cinar et al. 2008 | Lymphedema | RCT | 57 | Physiotherapist | Upper limb exercises | None | Not specified | 6-11 months | Intensive training during the initial period | Supervised program | Arm circumferences | Effective treatment† |
| Cornette et al. 2016 | CRF | RCT | 42 | Exercise expert | Combined exercise program (aerobic, resistance, flexbility, stretching, balance) | Periodic calls | 1-2 times/week | 6-11 months | Single initial consultation | Usual care | MFI, VO2, 6MWT | Effective treatment† |
|  | Bone, muscle, BMI | RCT | 42 | Exercise expert | Combined exercise program (aerobic, resistance, flexbility, stretching, balance) | Periodic calls | 1-2 times/week | 6-11 months | Single initial consultation | Usual care | Three-repetitions isometric bench, BMI | Effective treatment†* |
|  | QoL | RCT | 42 | Exercise expert | Combined exercise program (aerobic, resistance, flexbility, stretching, balance) | Periodic calls | 1-2 times/week | 6-11 months | Single initial consultation | Usual care | EORTC-QLQ-C30 | Effective treatment† |
|  | Cardiotoxicity | RCT | 42 | Exercise expert | Combined exercise program (aerobic, resistance, flexbility, stretching, balance) | Periodic calls | 1-2 times/week | 6-11 months | Single initial consultation | Usual care | VO2 maximum test, Heart rate, 6MWT | Effective treatment† |
| Courneya et al. 2007 | QoL | RCT | 242 | Exercise expert | Combined exercise program (aerobic, resistance, flexbility, stretching, balance) | None | Not specified | 6-11 months | Intensive training during the initial period | Usual care | FACT-An | Effective treatment† |
| Damush et al. 2006 | CRF | Single-arm trial | 34 | Nurse | Combined exercise program (aerobic, resistance, flexbility, stretching, balance) | None | ≥ 3 times/week | 6-11 months | Frequent contacts during the initial period | Not included | FACT-F, CHAMPS | Effective treatment† |
|  | QoL | Single-arm trial | 34 | Nurse | Combined exercise program (aerobic, resistance, flexbility, stretching, balance) | None | ≥ 3 times/week | 6-11 months | Frequent contacts during the initial period | Not included | FACT-F | Effective treatment† |
| Delrieu et al. 2020 | CRF | Single-arm trial | 49 | Exercise expert | Aerobic exercise program | Periodic calls | Daily | 6-11 months | Not delivered | Not included | PFS, EORT-QLQ-C30, 6MWT, VO2 | Ineffective treatment |
|  | Bone, muscle, BMI | Single-arm trial | 49 | Exercise expert | Aerobic exercise program | Periodic calls | Daily | 6-11 months | Not delivered | Not included | BMI, handgrip and back-leg dynamometer | Effective treatment† |
|  | QoL | Single-arm trial | 49 | Exercise expert | Aerobic exercise program | Periodic calls | Daily | 6-11 months | Not delivered | Not included | EORTC-QLQ—C30 | Ineffective treatment |
|  | Cardiotoxicity | Single-arm trial | 49 | Exercise expert | Aerobic exercise program | Periodic calls | Daily | 6-11 months | Not delivered | Not included | VO2 maximum test, 6MWT | Ineffective treatment |
| Demark-Wahnefried et al. 2008 | Bone, muscle, BMI | RCT | 90 | Others | Multidimensional program (physical, nutritional, physchological components) | Periodic calls | Daily | 6-11 months | Single initial consultation | Diet | BMI, BMD | Effective treatment† |
|  | QoL | RCT | 90 | Others | Multidimensional program (physical, nutritional, physchological components) | Periodic calls | Daily | 6-11 months | Single initial consultation | Diet | FACT-B | Effective treatment† |
| Demark-Wahnefried et al. 2015 | QoL | RCT | 692 | Not specified | Combined exercise program (aerobic, resistance, flexbility, stretching, balance) | Regular visits | ≥ 3 times/week | ≥ 1 year | Frequent contacts during the initial period | Usual care | SF-36, Impact of Cancer scale, Breast Cancer Prevention Trial Symptom Scale | Effective treatment† |
| Denysschen et al. 2011 | Bone, muscle, BMI | RCT | 100 | Exercise expert | Aerobic exercise program | Regular visits | Not specified | 3-5 months | Single initial consultation | Usual care | Fat and lean mass (DEXA) | Effective treatment† |
| DeNysschen et al. 2014 | Arthralgia | Single-arm trial | 26 | Exercise expert | Combined exercise program (aerobic, resistance, flexbility, stretching, balance) | Periodic calls | Daily | 1-2 months | Single initial consultation | Not included | AIMS2 | Effective treatment† |
|  | Bone, muscle, BMI | Single-arm trial | 26 | Exercise expert | Combined exercise program (aerobic, resistance, flexbility, stretching, balance) | Periodic calls | Daily | 1-2 months | Single initial consultation | Not included | Hand grip, Step test, Sit-to-stand | Effective treatment† |
|  | QoL | Single-arm trial | 26 | Exercise expert | Combined exercise program (aerobic, resistance, flexbility, stretching, balance) | Periodic calls | Daily | 1-2 months | Single initial consultation | Not included | SF-36 | Effective treatment† |
| Dieli-Conwright et al. 2018 | CRF | RCT | 100 | Exercise expert | Combined exercise program (aerobic, resistance, flexbility, stretching, balance) | None | ≥ 3 times/week | 3-5 months | Intensive training during the initial period | Usual care | BFI, VO2 | Effective treatment†* |
|  | Bone, muscle, BMI | RCT | 100 | Exercise expert | Combined exercise program (aerobic, resistance, flexbility, stretching, balance) | None | ≥ 3 times/week | 3-5 months | Intensive training during the initial period | Usual care | Muscle strength (leg, chest press 10 repetitions), BMD | Ineffective treatment |
|  | QoL | RCT | 100 | Exercise expert | Combined exercise program (aerobic, resistance, flexbility, stretching, balance) | None | ≥ 3 times/week | 3-5 months | Intensive training during the initial period | Usual care | FACT-B, SF-36 | Effective treatment†* |
|  | Cardiotoxicity | RCT | 100 | Exercise expert | Combined exercise program (aerobic, resistance, flexbility, stretching, balance) | None | ≥ 3 times/week | 3-5 months | Intensive training during the initial period | Usual care | VO2 maximum test | Effective treatment†* |
| Fields et al. 2016 | Arthralgia | Single-arm trial | 40 | Exercise expert | Aerobic exercise program | Periodic calls | 1-2 times/week | 1-2 months | Intensive training during the initial period | Not included | BPI | Effective treatment† |
|  | QoL | Single-arm trial | 40 | Exercise expert | Aerobic exercise program | Periodic calls | 1-2 times/week | 1-2 months | Intensive training during the initial period | Not included | SF-36 | Effective treatment† |
| Fillion et al. 2008 | CRF | RCT | 94 | Multidisciplinary team | Multidimensional program (physical, nutritional, physchological components) | Periodic calls | Not specified | 1-2 months | Intensive training during the initial period | Usual care | MFI | Effective treatment†* |
|  | Cardiotoxicity | RCT | 94 | Multidisciplinary team | Multidimensional program (physical, nutritional, physchological components) | Periodic calls | Not specified | 1-2 months | Intensive training during the initial period | Usual care | VO2 max | Effective treatment† |
|  | QoL | RCT | 94 | Multidisciplinary team | Multidimensional program (physical, nutritional, physchological components) | Periodic calls | Not specified | 1-2 months | Intensive training during the initial period | Usual care | SF-12, BPI | Effective treatment† |
| Fu et al. 2014 | Lymphedema | Single-arm trial | 140 | Nurse | Multidimensional program (physical, nutritional, physchological components) | None | Daily | ≥ 1 year | Single initial consultation | Not included | Perometry | Effective treatment† |
|  | Bone, muscle, BMI | Single-arm trial | 140 | Nurse | Multidimensional program (physical, nutritional, physchological components) | None | Daily | ≥ 1 year | Single initial consultation | Not included | BMI | Ineffective treatment |
| Galiano-Castillo et al. 2016 | CRF | RCT | 81 | Not specified | Combined exercise program (aerobic, resistance, flexbility, stretching, balance) | Periodic calls | ≥ 3 times/week | 1-2 months | Single initial consultation | Usual care | PFS | Effective treatment†* |
|  | Bone, muscle, BMI | RCT | 81 | Not specified | Combined exercise program (aerobic, resistance, flexbility, stretching, balance) | Periodic calls | ≥ 3 times/week | 1-2 months | Single initial consultation | Usual care | Handgrip dynamometer, back dynamometer, sit-to-stand | Effective treatment†* |
|  | QoL | RCT | 81 | Not specified | Combined exercise program (aerobic, resistance, flexbility, stretching, balance) | Periodic calls | ≥ 3 times/week | 1-2 months | Single initial consultation | Usual care | EORT-QLQ-C30, BPI | Effective treatment†* |
| Gautam et al. 2011 | Lymphedema | Single-arm trial | 38 | Physiotherapist | Complex decongestive therapy | Periodic calls | Daily | 1-2 months | Single initial consultation | Not included | Arm circumferences, volumetry | Effective treatment† |
|  | QoL | Single-arm trial | 38 | Physiotherapist | Complex decongestive therapy | Periodic calls | Daily | 1-2 months | Single initial consultation | Not included | SF-36 | Effective treatment† |
| Gencay Can et al. 2019 | Lymphedema | Single-arm trial | 25 | Not specified | Combined exercise program (aerobic, resistance, flexbility, stretching, balance) | None | Daily | 1-2 months | Single initial consultation | Not included | Arm circumferences, DASH | Effective treatment† |
|  | QoL | Single-arm trial | 25 | Not specified | Combined exercise program (aerobic, resistance, flexbility, stretching, balance) | None | Daily | 1-2 months | Single initial consultation | Not included | SF-36 | Effective treatment† |
| Gokal et al. 2016 | CRF | RCT | 50 | Not specified | Aerobic exercise program | None | ≥ 3 times/week | 3-5 months | Not delivered | Usual care | FACT-F, VO2, KPS | Effective treatment†* |
| Gregoire et al. 2017 | CRF | Four-arm trial | 123 | Mind-body teacher | Mind-body practice (yoga, qigong, tai chi) | None | Not specified | 6-11 months | Intensive training during the initial period | Physichological session / Usual care | EORTC-QLQ-C30 | Ineffective treatment |
| Harder et al. 2015 | Lymphedema | RCT | 92 | Multidisciplinary team | Mind-body practice (yoga, qigong, tai chi) | None | 1-2 times/week | 1-2 months | Single initial consultation | Usual care | Arm circumferences, DASH, Oxford shoulder score | Ineffective treatment |
|  | QoL | RCT | 92 | Multidisciplinary team | Mind-body practice (yoga, qigong, tai chi) | None | 1-2 times/week | 1-2 months | Single initial consultation | Usual care | FACT-B | Effective treatment† |
| Harvie et al. 2019 | Bone, muscle, BMI | RCT | 409 | Multidisciplinary team | Multidimensional program (physical, nutritional, physchological components) | Periodic calls | ≥ 3 times/week | 3-5 months | Single initial consultation | Supervised program | Body fat, body weight | Effective treatment† |
|  | QoL | RCT | 409 | Multidisciplinary team | Multidimensional program (physical, nutritional, physchological components) | Periodic calls | ≥ 3 times/week | 3-5 months | Single initial consultation | Supervised program | FACT | Effective treatment† |
| Huang et al. 2019 | CRF | RCT | 159 | Exercise expert | Aerobic exercise program | Periodic calls | ≥ 3 times/week | 3-5 months | Single initial consultation | Usual care | BFI | Effective treatment†* |
| Hughes et al. 2008 | Bone, muscle, BMI | Single-arm trial | 25 | Exercise expert | Combined exercise program (aerobic, resistance, flexbility, stretching, balance) | None | Not specified | 1-2 months | Single initial consultation | Not included | Muscle strength, Body fat percentage | Effective treatment† |
|  | QoL | Single-arm trial | 25 | Exercise expert | Combined exercise program (aerobic, resistance, flexbility, stretching, balance) | None | Not specified | 1-2 months | Single initial consultation | Not included | SF-36 | Ineffective treatment |
|  | Cardiotoxicity | Single-arm trial | 25 | Exercise expert | Combined exercise program (aerobic, resistance, flexbility, stretching, balance) | None | Not specified | 1-2 months | Single initial consultation | Not included | VO2 maximum test | Effective treatment† |
| Husebø et al. 2014 | CRF | RCT | 67 | Not specified | Combined exercise program (aerobic, resistance, flexbility, stretching, balance) | Periodic calls | Daily | 6-11 months | Not delivered | Usual care | SCFS-6, 6MWT | Ineffective treatment |
| Jacot et al. 2020 | CRF | RCT | 360 | Multidisciplinary team | Combined exercise program (aerobic, resistance, flexbility, stretching, balance) | None | 1-2 times/week | 6-11 months | Intensive training during the initial period | Usual care | MFI | Ineffective treatment |
|  | QoL | RCT | 360 | Multidisciplinary team | Combined exercise program (aerobic, resistance, flexbility, stretching, balance) | None | 1-2 times/week | 6-11 months | Intensive training during the initial period | Usual care | EORTC-QLQ-C30 | Ineffective treatment |
| Jeffs et al. 2013 | Lymphedema | Pilot study | 23 | Physiotherapist | Combined exercise program (aerobic, resistance, flexbility, stretching, balance) | None | Daily | 6-11 months | Single initial consultation | Usual care | Perometry, LYMQOL, DASH-9 | Effective treatment† |
| Johansson et al. 2013 | Lymphedema | Pilot study | 29 | Physiotherapist | Combined exercise program (aerobic, resistance, flexbility, stretching, balance) | None | ≥ 3 times/week | 1-2 months | Single initial consultation | Usual care | Perometry, bio-impedance spectroscopy, tissue dielectric constant | Ineffective treatment |
| Johansson et al. 2014 | Lymphedema | Single-arm trial | 23 | Physiotherapist | Resistance exercise program | None | ≥ 3 times/week | 1-2 months | Intensive training during the initial period | Not included | Volumetry, bio-impedance spectroscopy, DASH | Ineffective treatment |
| Kim et al. 2016 | Bone, muscle, BMI | Pilot study | 43 | Multidisciplinary team | Multidimensional program (physical, nutritional, physchological components) | Periodic calls | ≥ 3 times/week | 6-11 months | Single initial consultation | Diet | BMD, handgrip dynamometer, 30-sec Sit-to-stand, log | Ineffective treatment |
| Kim et al. 2019 | Bone, muscle, BMI | Pragmatic trial | 72 | Physiotherapist | Upper limb exercises | None | ≥ 3 times/week | 3-5 months | Single initial consultation | Active group | Hand grip dynamometer | Ineffective treatment |
|  | QoL | Pragmatic trial | 72 | Physiotherapist | Upper limb exercises | None | ≥ 3 times/week | 3-5 months | Single initial consultation | Active group | FACT-B, Shoulder Pain and Disability Index | Effective treatment† |
| Knobf et al. 2016 | Bone, muscle, BMI | RCT | 154 | Not specified | Multidimensional program (physical, nutritional, physchological components) | None | ≥ 3 times/week | 6-11 months | Intensive training during the initial period | Usual care | BMD (DEXA) | Ineffective treatment |
| Koch et al. 2017 | CRF | RCT | 40 | Mind-body teacher | Mind-body practice (yoga, qigong, tai chi) | None | Daily | 3-5 months | Intensive training during the initial period | Usual care | FACIT-F | Ineffective treatment |
|  | QoL | RCT | 40 | Mind-body teacher | Mind-body practice (yoga, qigong, tai chi) | None | Daily | 3-5 months | Intensive training during the initial period | Usual care | FACT-B | Ineffective treatment |
| Komatsu et al. 2016 | CRF | Single-arm trial | 18 | Mind-body teacher | Mind-body practice (yoga, qigong, tai chi) | None | Not specified | 1-2 months | Single initial consultation | Not included | CFS | Ineffective treatment |
|  | QoL | Single-arm trial | 18 | Mind-body teacher | Mind-body practice (yoga, qigong, tai chi) | None | Not specified | 1-2 months | Single initial consultation | Not included | CFS | Ineffective treatment |
| Lahart et al. 2016 | Bone, muscle, BMI | RCT | 80 | Exercise expert | Aerobic exercise program | Periodic calls | ≥ 3 times/week | 6-11 months | Single initial consultation | Usual care | BMI | Effective treatment†* |
|  | QoL | RCT | 80 | Exercise expert | Aerobic exercise program | Periodic calls | ≥ 3 times/week | 6-11 months | Single initial consultation | Usual care | FACT-B | Effective treatment†* |
| Lahart et al. 2018 | QoL | RCT | 32 | Nurse | Aerobic exercise program | Periodic calls | ≥ 3 times/week | 6-11 months | Single initial consultation | Usual care | BMI | Effective treatment† |
|  | Cardiotoxicity | RCT | 32 | Nurse | Aerobic exercise program | Periodic calls | ≥ 3 times/week | 6-11 months | Single initial consultation | Usual care | VO2 maximum test | Effective treatment† |
| Leclerc et al. 2018 | CRF | Pragmatic trial | 209 | Multidisciplinary team | Multidimensional program (physical, nutritional, physchological components) | None | ≥ 3 times/week | 3-5 months | Intensive training during the initial period | Usual care | FACIT-F | Effective treatment† |
|  | QoL | Pragmatic trial | 209 | Multidisciplinary team | Multidimensional program (physical, nutritional, physchological components) | None | ≥ 3 times/week | 3-5 months | Intensive training during the initial period | Usual care | EORTC-QLQ-C30, EQ-5D | Effective treatment† |
| Letellier et al. 2014 | Lymphedema | Pilot study | 25 | Physiotherapist | Complex decongestive therapy | None | Daily | 3-5 months | Not delivered | Supervised program | Arm circumferences, arm volume, DASH, McGill Pain Questionnaire | Ineffective treatment |
|  | QoL | Pilot study | 25 | Physiotherapist | Complex decongestive therapy | None | Daily | 3-5 months | Not delivered | Supervised program | FACT-B | Ineffective treatment |
| Ligabue et al. 2019 | Lymphedema | RCT | 41 | Physiotherapist | Complex decongestive therapy | None | Daily | 6-11 months | Intensive training during the initial period | Usual care | Volumetry, VAS pain | Effective treatment†* |
| Ligibel et al. 2010 | Bone, muscle, BMI | Single-arm trial | 41 | Exercise expert | Aerobic exercise program | Periodic calls | ≥ 3 times/week | 3-5 months | Single initial consultation | Not included | BMI | Ineffective treatment |
|  | QoL | Single-arm trial | 41 | Exercise expert | Aerobic exercise program | Periodic calls | ≥ 3 times/week | 3-5 months | Single initial consultation | Not included | EORTC-QLQ-C30 | Effective treatment† |
|  | Cardiotoxicity | Single-arm trial | 41 | Exercise expert | Aerobic exercise program | Periodic calls | ≥ 3 times/week | 3-5 months | Single initial consultation | Not included | VO2 maximum test | Effective treatment† |
| Liu et al. 2021 | Lymphedema | Single-arm trial | 41 | Not specified | Aerobic exercise program | Periodic calls | Daily | ≥ 1 year | Single initial consultation | Not included | Arm circumferences, Breast Cancer and Lymphedema Symptom Experience Index | Effective treatment† |
| Loh et al. 2014 | CRF | RCT | 197 | Mind-body teacher | Mind-body practice (yoga, qigong, tai chi) | None | Not specified | 6-11 months | Intensive training during the initial period | Active group | FACIT-F | Effective treatment† |
| Loudon et al. 2014 | Lymphedema | RCT | 28 | Mind-body teacher | Multidimensional program (physical, nutritional, physchological components) | Periodic calls | Daily | 1-2 months | Intensive training during the initial period | Usual care | Arm circumference, bioimpedance, VAS pain | Ineffective treatment |
|  | QoL | RCT | 28 | Mind-body teacher | Multidimensional program (physical, nutritional, physchological components) | Periodic calls | Daily | 1-2 months | Intensive training during the initial period | Usual care | LYMQOL | Ineffective treatment |
| Lotzke et al. 2016 | CRF | RCT | 119 | Mind-body teacher | Mind-body practice (yoga, qigong, tai chi) | None | 1-2 times/week | 3-5 months | Intensive training during the initial period | Active group | Cancer Fatigue Scale | Ineffective treatment |
|  | QoL | RCT | 119 | Mind-body teacher | Mind-body practice (yoga, qigong, tai chi) | None | 1-2 times/week | 3-5 months | Intensive training during the initial period | Active group | EORT-QLQ-C30 | Effective treatment† |
| Mascherini et al. 2019 | Bone, muscle, BMI | Single-arm trial | 43 | Not specified | Aerobic exercise program | Regular visits | ≥ 3 times/week | ≥ 1 year | Not delivered | Not included | BMI, skinfold thickness, bioimpedance, handgrip test, chair test | Effective treatment† |
| Mascherini et al. 2020 | Bone, muscle, BMI | Single-arm trial | 42 | Exercise expert | Combined exercise program (aerobic, resistance, flexbility, stretching, balance) | None | ≥ 3 times/week | 6-11 months | Single initial consultation | Not included | BMI, handgrip, chair test | Effective treatment† |
|  | QoL | Single-arm trial | 42 | Exercise expert | Combined exercise program (aerobic, resistance, flexbility, stretching, balance) | None | ≥ 3 times/week | 6-11 months | Single initial consultation | Not included | SF-36 | Effective treatment† |
|  | Cardiotoxicity | Single-arm trial | 42 | Exercise expert | Combined exercise program (aerobic, resistance, flexbility, stretching, balance) | None | ≥ 3 times/week | 6-11 months | Single initial consultation | Not included | 6MWT, heart rate, pressure values | Effective treatment† |
| Matthews et al. 2006 | Bone, muscle, BMI | RCT | 36 | Not specified | Aerobic exercise program | Periodic calls | ≥ 3 times/week | 3-5 months | Single initial consultation | Usual care | BMI, BMD | Ineffective treatment |
| McNeil et al. 2019 | Bone, muscle, BMI | RCT | 45 | Exercise expert | Aerobic exercise program | None | ≥ 3 times/week | 3-5 months | Not delivered | Usual care | BMI | Effective treatment† |
|  | Cardiotoxicity | RCT | 45 | Exercise expert | Aerobic exercise program | None | ≥ 3 times/week | 3-5 months | Not delivered | Usual care | VO2 maximum test | Effective treatment†* |
| Mijwel et al. 2019 | CRF | RCT | 240 | Multidisciplinary team | Combined exercise program (aerobic, resistance, flexbility, stretching, balance) | Regular visits | Not specified | 6-11 months | Intensive training during the initial period | Usual care | PFS, VO2 | Effective treatment†* |
|  | Bone, muscle, BMI | RCT | 240 | Multidisciplinary team | Combined exercise program (aerobic, resistance, flexbility, stretching, balance) | Regular visits | Not specified | 6-11 months | Intensive training during the initial period | Usual care | BMI, mid-thigh pull, handgrip dynamometer | Effective treatment†* |
|  | QoL | RCT | 240 | Multidisciplinary team | Combined exercise program (aerobic, resistance, flexbility, stretching, balance) | Regular visits | Not specified | 6-11 months | Intensive training during the initial period | Usual care | EORTC-QLQ-C30 | Effective treatment† |
|  | Cardiotoxicity | RCT | 240 | Multidisciplinary team | Combined exercise program (aerobic, resistance, flexbility, stretching, balance) | Regular visits | Not specified | 6-11 months | Intensive training during the initial period | Usual care | VO2 maximum test | Effective treatment† |
| Mirolo et al. 1995 | Lymphedema | Single-arm trial | 25 | Nurse | Complex decongestive therapy | None | Not specified | 1-2 months | Intensive training during the initial period | Not included | Arm circumferences | Effective treatment† |
|  | QoL | Single-arm trial | 25 | Nurse | Complex decongestive therapy | None | Not specified | 1-2 months | Intensive training during the initial period | Not included | Functional Living Index -cancer | Ineffective treatment |
| Moattari et al. 2012 | Lymphedema | Single-arm trial | 21 | Not specified | Complex decongestive therapy | Periodic calls | Daily | 1-2 months | Intensive training during the initial period | Not included | Volumetry, arm circumferences, shoulder range of motion | Effective treatment† |
| Mock et al. 2001 | CRF | RCT | 52 | Not specified | Aerobic exercise program | Periodic calls | 1-2 times/week | 3-5 months | Single initial consultation | Usual care | PFS, 12MWT | Effective treatment†* |
|  | QoL | RCT | 52 | Not specified | Aerobic exercise program | Periodic calls | 1-2 times/week | 3-5 months | Single initial consultation | Usual care | MOS-SF36 | Effective treatment†* |
| Mock et al. 2005 | CRF | RCT | 119 | Nurse | Aerobic exercise program | Periodic calls | ≥ 3 times/week | 1-2 months | Single initial consultation | Usual care | PFS, 12MWT | Ineffective treatment |
| Møller et al. 2020 | Bone, muscle, BMI | RCT | 153 | Nurse | Aerobic exercise program | Regular visits | ≥ 3 times/week | 3-5 months | Not delivered | Supervised program | BMI, dynamometer (lower limb), BMD | Ineffective treatment |
|  | QoL | RCT | 153 | Nurse | Aerobic exercise program | Regular visits | ≥ 3 times/week | 3-5 months | Not delivered | Supervised program | EORTC-QLQ | Ineffective treatment |
|  | Cardiotoxicity | RCT | 153 | Nurse | Aerobic exercise program | Regular visits | ≥ 3 times/week | 3-5 months | Not delivered | Supervised program | VO2 maximum test | Ineffective treatment |
| Musanti 2012 | Bone, muscle, BMI | RCT | 42 | Not specified | Combined exercise program (aerobic, resistance, flexbility, stretching, balance) | Periodic calls | Not specified | 3-5 months | Single initial consultation | Active group | BMI, chest, bench, leg press | Effective treatment† |
|  | Cardiotoxicity | RCT | 42 | Not specified | Combined exercise program (aerobic, resistance, flexbility, stretching, balance) | Periodic calls | Not specified | 3-5 months | Single initial consultation | Active group | VO2 maximum test | Ineffective treatment |
|  | CRF | RCT | 42 | Not specified | Combined exercise program (aerobic, resistance, flexbility, stretching, balance) | Periodic calls | Not specified | 3-5 months | Single initial consultation | Active group | PFS | Ineffective treatment |
| Noura et al. 2021 | Lymphedema | Non-randomized trial | 60 | Not specified | Complex decongestive therapy | None | Not specified | 3-5 months | Intensive training during the initial period | Usual care | DASH, McGill Pain Questionnaire | Effective treatment†* |
| Nyrop et al. 2017 | Arthralgia | RCT | 62 | Not specified | Aerobic exercise program | None | ≥ 3 times/week | 1-2 months | Not delivered | Usual care | VAS, WOMAC, RAI | Effective treatment† |
|  | CRF | RCT | 62 | Not specified | Aerobic exercise program | None | ≥ 3 times/week | 1-2 months | Not delivered | Usual care | Visual Analogue Scale | Ineffective treatment |
| Ochi et al. 2021 | CRF | RCT | 50 | Not specified | Aerobic exercise program | Phone notifications | ≥ 3 times/week | 3-5 months | Not delivered | Usual care | Cancer Fatigue Scale, VO2, 6MWT | Effective treatment† |
|  | Bone, muscle, BMI | RCT | 50 | Not specified | Aerobic exercise program | Phone notifications | ≥ 3 times/week | 3-5 months | Not delivered | Usual care | 1-repetition maximum test (leg press, grip strength, chair stand test) | Effective treatment† |
|  | QoL | RCT | 50 | Not specified | Aerobic exercise program | Phone notifications | ≥ 3 times/week | 3-5 months | Not delivered | Usual care | EQ-5D | Ineffective treatment |
|  | Cardiotoxicity | RCT | 50 | Not specified | Aerobic exercise program | Phone notifications | ≥ 3 times/week | 3-5 months | Not delivered | Usual care | VO2 maximum test, 6MWT | Effective treatment†* |
| Paskett et al. 2021 | Lymphedema | RCT | 554 | Physiotherapist | Combined exercise program (aerobic, resistance, flexbility, stretching, balance) | Periodic calls | Daily | ≥ 1 year | Single initial consultation | Usual care | Arm circumferences, interlimb volume, shoulder ROMs | Ineffective treatment |
| Payne et al. 2008 | CRF | Pilot study | 20 | Nurse | Aerobic exercise program | None | ≥ 3 times/week | 3-5 months | Not delivered | Usual care | PFS | Ineffective treatment |
| Pinto et al. 2005 | CRF | RCT | 86 | Not specified | Aerobic exercise program | Periodic calls | ≥ 3 times/week | 3-5 months | Not delivered | Usual care | Linear analogue scale for CFR | Effective treatment†* |
|  | Bone, muscle, BMI | RCT | 86 | Not specified | Aerobic exercise program | Periodic calls | ≥ 3 times/week | 3-5 months | Not delivered | Usual care | BMI | Ineffective treatment |
| Pinto et al. 2008 | CRF | RCT | 86 | Not specified | Aerobic exercise program | Periodic calls | Not specified | 6-11 months | Frequent contacts during the initial period | Usual care | Linear analogue scale for fatigue | Effective treatment†* |
| Porter et al. 2019 | CRF | Pilot study | 63 | Mind-body teacher | Mind-body practice (yoga, qigong, tai chi) | None | Daily | 3-5 months | Intensive training during the initial period | Physichological sessions | BFI, 6MWT, BPI | Effective treatment† |
| Prieto-Gomez et al. 2022 | CRF | RCT | 80 | Physiotherapist | Aerobic exercise program | Education/support sessions | ≥ 3 times/week | 1-2 months | Single initial consultation | Supervised program | FACIT-F, 6MWT, VAS pain | Ineffective treatment |
| Qiao et al. 2022 | CRF | RCT | 60 | Exercise expert | Aerobic exercise program | Regular visits | ≥ 3 times/week | 3-5 months | Single initial consultation | Usual care | FACT-ES, Pittsburgh fatigability | Ineffective treatment |
| Randheer et al. 2011 | Lymphedema | Observational study | 25 | Physiotherapist | Complex decongestive therapy | None | Daily | 3-5 months | Intensive training during the initial period | Not included | Volumetry | Effective treatment† |
| Rogers et al. 2013 | CRF | RCT | 28 | Multidisciplinary team | Combined exercise program (aerobic, resistance, flexbility, stretching, balance) | Education/support sessions | ≥ 3 times/week | 1-2 months | Intensive training during the initial period | Usual care | FSI | Ineffective treatment |
|  | Bone, muscle, BMI | RCT | 28 | Multidisciplinary team | Combined exercise program (aerobic, resistance, flexbility, stretching, balance) | Education/support sessions | ≥ 3 times/week | 1-2 months | Intensive training during the initial period | Usual care | BMI, back-leg dynamometer | Ineffective treatment |
| Rogers et al. 2015 | QoL | RCT | 222 | Exercise expert | Aerobic exercise program | Education/support sessions | ≥ 3 times/week | 1-2 months | Intensive training during the initial period | Usual care | FACT-B | Effective treatment†* |
| Schmitz et al. 2010 | Lymphedema | RCT | 134 | Exercise expert | Resistance exercise program | None | 1-2 times/week | 6-11 months | Intensive training during the initial period | Usual care | Volumetry, one-repetition maximum test (bench and leg press), symptom questionnaire | Effective treatment† |
| Schmitz et al. 2019 | Lymphedema | RCT | 351 | Exercise expert | Multidimensional program (physical, nutritional, physchological components) | Periodic calls | 1-2 times/week | 6-11 months | Intensive training during the initial period | Usual care | Interlimb volume difference, clinical characteristics of lymphedema, Norman Lymphedema Survey | Effective treatment† |
|  | Bone, muscle, BMI | RCT | 351 | Exercise expert | Multidimensional program (physical, nutritional, physchological components) | Periodic calls | 1-2 times/week | 6-11 months | Intensive training during the initial period | Usual care | Bench and leg press, body weight | Effective treatment†* |
|  | Cardiotoxicity | RCT | 351 | Exercise expert | Multidimensional program (physical, nutritional, physchological components) | Periodic calls | 1-2 times/week | 6-11 months | Intensive training during the initial period | Usual care | VO2 maximum test | Effective treatment† |
| Schröder et al. 2022 | CRF | RCT | 51 | Multidisciplinary team | Aerobic exercise program | None | Not specified | 1-2 months | Intensive training during the initial period | Active group | Cancer Fatigue Scale | Ineffective treatment |
|  | QoL | RCT | 51 | Multidisciplinary team | Aerobic exercise program | None | Not specified | 1-2 months | Intensive training during the initial period | Active group | WHOQOL-BREF, FACT-G, NRS pain | Ineffective treatment |
| Schwartz 2000 | CRF | Pragmatic trial | 31 | Not specified | Aerobic exercise program | None | ≥ 3 times/week | 1-2 months | Not delivered | Usual care | Linear analogue scale for CRF, 12MWT | Effective treatment†* |
| Schwartz 2000 (2) | CRF | Pragmatic trial | 78 | Not specified | Aerobic exercise program | Periodic calls | ≥ 3 times/week | 1-2 months | Single initial consultation | Usual care | SCFS-6 | Effective treatment†* |
| Schwartz et al. 2001 | CRF | Single-arm trial | 72 | Not specified | Aerobic exercise program | Periodic calls | ≥ 3 times/week | 1-2 months | Not delivered | Not included | Linear analogue scale for CRF, 12MWT | Effective treatment† |
| Spector et al. 2014 | CRF | Single-arm trial | 17 | Exercise expert | Combined exercise program (aerobic, resistance, flexbility, stretching, balance) | Periodic calls | ≥ 3 times/week | 3-5 months | Single initial consultation | Not included | FACIT-F, VO2 | Ineffective treatment |
|  | Bone, muscle, BMI | Single-arm trial | 17 | Exercise expert | Combined exercise program (aerobic, resistance, flexbility, stretching, balance) | Periodic calls | ≥ 3 times/week | 3-5 months | Single initial consultation | Not included | Dual energy X-ray absorptiometry, dynamometer (quadriceps, rhomboid major and minor) | Ineffective treatment |
|  | QoL | Single-arm trial | 17 | Exercise expert | Combined exercise program (aerobic, resistance, flexbility, stretching, balance) | Periodic calls | ≥ 3 times/week | 3-5 months | Single initial consultation | Not included | FACT-B, Selective Functional Movement Assessment | Ineffective treatment |
|  | Cardiotoxicity | Single-arm trial | 17 | Exercise expert | Combined exercise program (aerobic, resistance, flexbility, stretching, balance) | Periodic calls | ≥ 3 times/week | 3-5 months | Single initial consultation | Not included | VO2 maximum test | Effective treatment† |
| Stan et al. 2016 | CRF | RCT | 34 | Multidisciplinary team | MInd-body practice (yoga, qigong, tai chi) | None | ≥ 3 times/week | 3-5 months | Not delivered | Active group | MFSI-SF | Effective treatment† |
|  | QoL | RCT | 34 | Multidisciplinary team | MInd-body practice (yoga, qigong, tai chi) | None | ≥ 3 times/week | 3-5 months | Not delivered | Active group | FACT-B | Effective treatment† |
| Sturgeon et al. 2022 | CRF | RCT | 19 | Exercise expert | Aerobic exercise program | Periodic calls | ≥ 3 times/week | 6-11 months | Not delivered | Usual care | MFSI-SF | Effective treatment†* |
|  | Cardiotoxicity | RCT | 19 | Exercise expert | Aerobic exercise program | Periodic calls | ≥ 3 times/week | 6-11 months | Not delivered | Usual care | VO2 max | Effective treatment†* |
|  | QoL | RCT | 19 | Exercise expert | Aerobic exercise program | Periodic calls | ≥ 3 times/week | 6-11 months | Not delivered | Usual care | SF-36 (pain, emotional, physical domains) | Effective treatment†* |
| Swenson et al. 2009 | CRF | RCT | 72 | Physiotherapist | Multidimensional program (physical, nutritional, physchological components) | Regular visits | Daily | ≥ 1 year | Single initial consultation | Diet | SCFS, MDASI | Effective treatment† |
|  | Bone, muscle, BMI | RCT | 72 | Physiotherapist | Multidimensional program (physical, nutritional, physchological components) | Regular visits | Daily | ≥ 1 year | Single initial consultation | Diet | BMD (Absorbiometry DEXA) | Ineffective treatment |
| Temur et al. 2019 | Lymphedema | RCT | 61 | Nurse | Complex decongestive therapy | Periodic calls | Daily | 6-11 months | Intensive training during the initial period | Usual care | DASH, arm circumferences | Effective treatment†* |
|  | QoL | RCT | 61 | Nurse | Complex decongestive therapy | Periodic calls | Daily | 6-11 months | Intensive training during the initial period | Usual care | EORTC-QLQ-30, EORTC-QLQ-BR23 | Effective treatment†* |
| Todd et al. 2008 | Lymphedema | RCT | 116 | Nurse | Upper limb exercises | Periodic calls | Daily | ≥ 1 year | Intensive training during the initial period | Active group | Volumetry, DASH | Ineffective treatment |
|  | QoL | RCT | 116 | Nurse | Upper limb exercises | Periodic calls | Daily | ≥ 1 year | Intensive training during the initial period | Active group | FACT-B | Ineffective treatment |
| Van Waart et al. 2015 | CRF | Three-arm trial | 230 | Nurse | Aerobic exercise program | None | ≥ 3 times/week | 3-5 months | Single initial consultation | Supervised program / usual care | MFI, Fatigue Quality List | Ineffective treatment |
|  | Bone, muscle, BMI | Three-arm trial | 230 | Nurse | Aerobic exercise program | None | ≥ 3 times/week | 3-5 months | Single initial consultation | Supervised program / usual care | Handheld dynamometer (grip, elbow flexion, knee extension), 30-s chair stand test | Ineffective treatment |
|  | QoL | Three-arm trial | 230 | Nurse | Aerobic exercise program | None | ≥ 3 times/week | 3-5 months | Single initial consultation | Supervised program / usual care | EORTC-QLQ-C30 | Effective treatment† |
|  | Cardiotoxicity | Three-arm trial | 230 | Nurse | Aerobic exercise program | None | ≥ 3 times/week | 3-5 months | Single initial consultation | Supervised program / usual care | Steep ramp test | Ineffective treatment |
| VanderWalde et al. 2021 | CRF | RCT | 54 | Not specified | Aerobic exercise program | None | ≥ 3 times/week | 1-2 months | Not delivered | Active group | FSI, PROMIS Fatigue, PROMIS pain, SFPB | Ineffective treatment |
| Vignes et al. 2007 | Lymphedema | Observational study | 537 | Physiotherapist | Complex decongestive therapy | Regular visits | ≥ 3 times/week | ≥ 1 year | Intensive training during the initial period | Not included | Truncated cone | Effective treatment† |
| Vincent et al. 2013 | CRF | Single-arm trial | 34 | Physiotherapist | Aerobic exercise program | Periodic calls | ≥ 3 times/week | 3-5 months | Not delivered | Not included | PFS-R, 6MWT, VO2 | Ineffective treatment |
|  | Cardiotoxicity | Single-arm trial | 34 | Physiotherapist | Aerobic exercise program | Periodic calls | ≥ 3 times/week | 3-5 months | Not delivered | Not included | VO2 maximum test, 6MWT | Effective treatment† |
| Vincent et al. 2020 | CRF | RCT | 81 | Exercise expert | Combined exercise program (aerobic, resistance, flexbility, stretching, balance) | Periodic calls | 1-2 times/week | ≥ 1 year | Single initial consultation | Active group | MFI, 6MWT, VO2 | Ineffective treatment |
|  | Bone, muscle, BMI | RCT | 81 | Exercise expert | Combined exercise program (aerobic, resistance, flexbility, stretching, balance) | Periodic calls | 1-2 times/week | ≥ 1 year | Single initial consultation | Active group | BMI, 3-repetition bench (lower limb) | Effective treatment† |
|  | QoL | RCT | 81 | Exercise expert | Combined exercise program (aerobic, resistance, flexbility, stretching, balance) | Periodic calls | 1-2 times/week | ≥ 1 year | Single initial consultation | Active group | EORTC-QLQ-C30 | Ineffective treatment |
|  | Cardiotoxicity | RCT | 81 | Exercise expert | Combined exercise program (aerobic, resistance, flexbility, stretching, balance) | Periodic calls | 1-2 times/week | ≥ 1 year | Single initial consultation | Active group | VO2 maximum test, 6MWT | Effective treatment† |
| Waltman et al. 2003 | Bone, muscle, BMI | Single-arm trial | 21 | Not specified | Multidimensional program (physical, nutritional, physchological components) | Periodic calls | Not specified | ≥ 1 year | Not delivered | Not included | Muscle strength, BMD | Effective treatment† |
| Westphal et al. 2019 | Bone, muscle, BMI | RCT | 50 | Not specified | Combined exercise program (aerobic, resistance, flexbility, stretching, balance) | None | 1-2 times/week | 6-11 months | Intensive training during the initial period | Active group | BMI, body fat | Ineffective treatment |
|  | QoL | RCT | 50 | Not specified | Combined exercise program (aerobic, resistance, flexbility, stretching, balance) | None | 1-2 times/week | 6-11 months | Intensive training during the initial period | Active group | EORTC-QLQ-C30, EORTC-QLQ-BR23 | Ineffective treatment |
| Winters-Stone et al. 2017 | CRF | RCT | 95 | Physiotherapist | Mind-body practice (yoga, qigong, tai chi) | None | ≥ 3 times/week | 1-2 months | Not delivered | Education sessions | POMS-B | Effective treatment†* |
| Winters-Stone et al. 2021 | Bone, muscle, BMI | RCT | 114 | Exercise expert | Combined exercise program (aerobic, resistance, flexbility, stretching, balance) | Phone notifications | ≥ 3 times/week | 6-11 months | Intensive training during the initial period | Active group | 1-repetition maximum test (leg and chest press) | Ineffective treatment |
|  | QoL | RCT | 114 | Exercise expert | Combined exercise program (aerobic, resistance, flexbility, stretching, balance) | Phone notifications | ≥ 3 times/week | 6-11 months | Intensive training during the initial period | Active group | SF-36 | Ineffective treatment |
| Wonders et al. 2013 | QoL | Single-arm trial | 6 | Not specified | Combined exercise program (aerobic, resistance, flexbility, stretching, balance) | Phone notifications | ≥ 3 times/week | 1-2 months | Single initial consultation | Not included | McGill QoL questionnaire | Effective treatment† |
|  | Neurotoxicity | Single-arm trial | 6 | Not specified | Combined exercise program (aerobic, resistance, flexbility, stretching, balance) | Phone notifications | ≥ 3 times/week | 1-2 months | Single initial consultation | Not included | Leeds Assessment of Neuropathic Symptoms and Signs | Effective treatment† |
| Xu et al. 2021 | Bone, muscle, BMI | Single-arm trial | 109 | Exercise expert | Aerobic exercise program | Periodic calls | ≥ 3 times/week | 3-5 months | Single initial consultation | Not included | BMI, bioelectrical impedance | Effective treatment† |
|  | QoL | Single-arm trial | 109 | Exercise expert | Aerobic exercise program | Periodic calls | ≥ 3 times/week | 3-5 months | Single initial consultation | Not included | FACT-B | Ineffective treatment |
| Yang et al. 2011 | CRF | RCT | 40 | Not specified | Aerobic exercise program | Periodic calls | ≥ 3 times/week | 3-5 months | Not delivered | Usual care | MDASI-T | Effective treatment†* |
| Yuen et al. 2007 | CRF | Three-arm trial | 22 | Physiotherapist | Combined exercise program (aerobic, resistance, flexbility, stretching, balance) | Periodic calls | ≥ 3 times/week | 3-5 months | Not delivered | Active group / Usual care | PFS, fatigue calendars, 6MWT | Ineffective treatment |

* statistically significant between-groups difference (compared to the control group): the effect of self-managed PA is greater than that of the control treatment; † statistically significant within-group difference in the intervention group (compared to baseline values): the effect of self-managed PA is equal to that of the control treatment.
